# Uro-oncologic patient management during the COVID-19 pandemic: survey findings from an Italian oncologic hub

**DOI:** 10.2217/fon-2021-0145

**Published:** 2021-07-19

**Authors:** Stefano Luzzago, Francesco A Mistretta, Enza Dossena, Gianna Comandi, Giovanni Petralia, Dario Di Trapani, Gabriele Cozzi, Antonio Galfano, Matteo Ferro, Aldo M Bocciardi, Gennaro Musi, Ottavio de Cobelli

**Affiliations:** ^1^Department of Urology, IEO European Institute of Oncology, IRCCS, Via Ripamonti 435, Milan, Italy; ^2^Università degli Studi di Milano, Milan, Italy; ^3^Nurse Director, Multidisciplinary Surgical Area, European Institute of Oncology, IRCCS, Milan, 20100, Italy; ^4^Department of Urology, ASST Grande Ospedale Metropolitano Niguarda, Milan, Italy; ^5^Università degli Studi di Milano, Department of Oncology & Hematology-Oncology, Milan, Italy

**Keywords:** COVID-19, oncology service, SARS-CoV-2, surgical oncology, urological cancer

## Abstract

**Aim:** Patient and worker satisfaction at an oncologic hub during the COVID-19 pandemic has never been reported. We addressed this topic. **Methods:** We conducted a survey to test the views of patients (n = 64) and healthcare professionals (n = 52) involved with our operative protocol. **Results:** A moderate/severe grade of concern due to the COVID-19 emergency was recorded in 63% of patients versus 75% of hospital staff. High/very high versus low satisfaction grade about preventive strategies to reduce the risk of SARS-CoV-2 contagion was identified in the patients compared with the hospital staff group. **Conclusion:** Surgical treatment at a hub center of uro-oncologic patients coming from spoke centers is well accepted and should, therefore, be recommended. Preventive strategies to reduce the risk of SARS-CoV-2 contagion in hospital staff members should be implemented.

## Lay abstract

We provide robust evidence that an oncologic hub center during COVID-19 pandemic represents a credible solution for management of non-deferrable uro-oncologic patients. Specifically, surgical treatment at a hub center of patients coming from spoke centers is well accepted by both patients and hospital staff members. Moreover, collaboration between healthcare workers from spoke and hub centers generates minimal levels of anxiety, while potentially being associated with clinical, surgical and scientific improvement. This said, a more specific focus on recommended strategies to reduce the risk of SARS-CoV-2 contagion at oncologic hub hospitals is warranted.

The SARS-CoV-2 virus and its associated disease, COVID-19, have been recognized by WHO as a pandemic [1]. Several protocols to manage all non-deferrable oncologic and benign conditions, while simultaneously protecting the safety of healthcare professionals and patients, have been developed worldwide [2–5]. Specifically, on 8 March 2020, the Italian National Healthcare system organized specific oncologic hospitals, namely oncologic hubs, that have to provide care to all non-deferrable cancer patients [6]. To the best of our knowledge, no previous authors have addressed patients’ and workers’ satisfaction with regard to regional intervention models.

We recently published a specific guide to organize an oncologic hub hospital [6]. Here we present the results of an internal survey that was conducted to test patient and healthcare professionals’ involvement with our strict protocol and their satisfaction rate. Specifically, we focused on all non-deferrable uro-oncologic patients [7] who were treated at our oncologic hub center (European Institute of Oncology; IEO) during the COVID-19 pandemic.

## Patients & methods

### Study design & ethical considerations

We retrospectively analyzed all prospectively collected questionnaires that were administered to all patients treated at our center and to all hospital staff members involved in patient care between 16 March and 1 June 2020. The study design was approved by the Institutional Review Board of the IEO, Milan and was compliant with the Guidelines of the Declaration of Helsinki.

### Oncologic hub guidelines: preventive strategies

In order to treat non-deferrable cancer patients, the Italian healthcare system created oncologic hub hospitals, where strict protocols to maintain ‘COVID-free’ surgical and onco-hematologic wards were applied [6]. Due to the scarce availability of nasopharyngeal tests at the beginning of the pandemic, some simple strategies to reduce the risk of SARS-CoV-2 were adopted. For ambulatory patients, these were: telephone interviews before access to institution; preliminary triage at the entrance; itineraries and dedicated areas for specific tasks; only one companion allowed and only for non-self-sufficient patients; access to hospital comfort services limited to hospital staff. For in-hospital surgical patients, white blood cell count, C-reactive protein and lactate dehydrogenase blood levels were investigated in all patients within 5 days before surgery. In addition, chest radiography was performed in all patients whose preoperative examinations were conducted 30 or more days before surgery. Moreover, on the hospitalization day, the patient underwent a double check triage, at the hospital entrance and in the surgical ward. Last, visits to hospitalized patients were reduced as much as possible. For medical and paramedic staff, temperature was evaluated day by day before entering the hospital. During either daily clinic or desk activities, all hospital staff used adequate individual protection devices. However, due to limited availability of individual protection devices, the use of these devices was optimized. In consequence, strict individual protections (FFP2/FFP3 masks, two pairs of gloves, disposable cap, protective glasses or visor and disposable water-repellent gown) were available only for healthcare workers exposed to higher risk of contagion (aerosol or droplet emission risk procedures, COVID-19 ward etc.). Phone or web conferences were implemented. Last, dedicated areas for both suspected and confirmed COVID-19 patients were built, where only deputed multidisciplinary medical and paramedic personnel had access.

### Study population: patient management at the oncologic hub

Overall, 377 non-deferrable uro-oncologic patients were treated during the study period (16 March–1 June 2020). Specifically, surgical/perioperative management of patients included the following: patients coming from the spoke center (Grande Ospedale Metropolitano Niguarda [H Niguarda]) with shared management between spoke (H Niguarda) and hub (IEO) centers (47 patients, 12.5%); patients coming from other spoke centers with exclusive management by the hub center (IEO) (43 patients, 11.5%); patients coming from the hub center (IEO) with exclusive management by the hub center (IEO) (287 patients, 76%). Because no validated questionnaires were available during the study period, a *de novo* survey was created for the purpose of the analysis. A 15-item survey (Supplementary Figure 1) was administered to all patients in categories 1 and 2, 1 month after surgery. Category 3 patients were not considered for the analysis, because these subjects had their surgery already scheduled at the beginning of the ‘hub project’. Overall, 33 (70.5%) and 31 (72%) patients of category 1 and category 2 completed the survey and were available for final analysis. Patients who refused to complete the questionnaire, as well as those who were lost to follow-up, were excluded (n = 26; 29%).

### Study population: hospital staff members

Overall, 52 hospital staff members were involved in patient management during the study period. Again, because no validated questionnaires were available during the study period, a *de novo* survey was created for the purpose of the analysis. Different questionnaires were administered to ward or scrub nurses and to physicians. Specifically, a 16-item survey (Supplementary Figure 2) was completed by both ward (n = 18) and scrub nurses (n = 9) coming from the hub center (IEO), while a 21-item questionnaire was completed by physicians coming from the spoke (H Niguarda; n = 7) or hub (IEO; n = 18) centers (Supplementary Figure 3).

### Analyses & outcomes

For the purpose of these analyses, patients and hospital staff members were asked to evaluate five major outcomes: impact of the COVID-19 emergency on daily life and clinical practice; patient satisfaction after treatment at the hub center (IEO); preventive strategies to reduce the risk of SARS-CoV-2 contagion; collaboration between spoke (H Niguarda) and hub center (IEO) staff; final considerations and model reproducibility. Frequencies and percentages were reported for all considered outcomes.

## Results

### Impact of COVID-19 emergency on daily life & clinical practice

**Figure 1.**
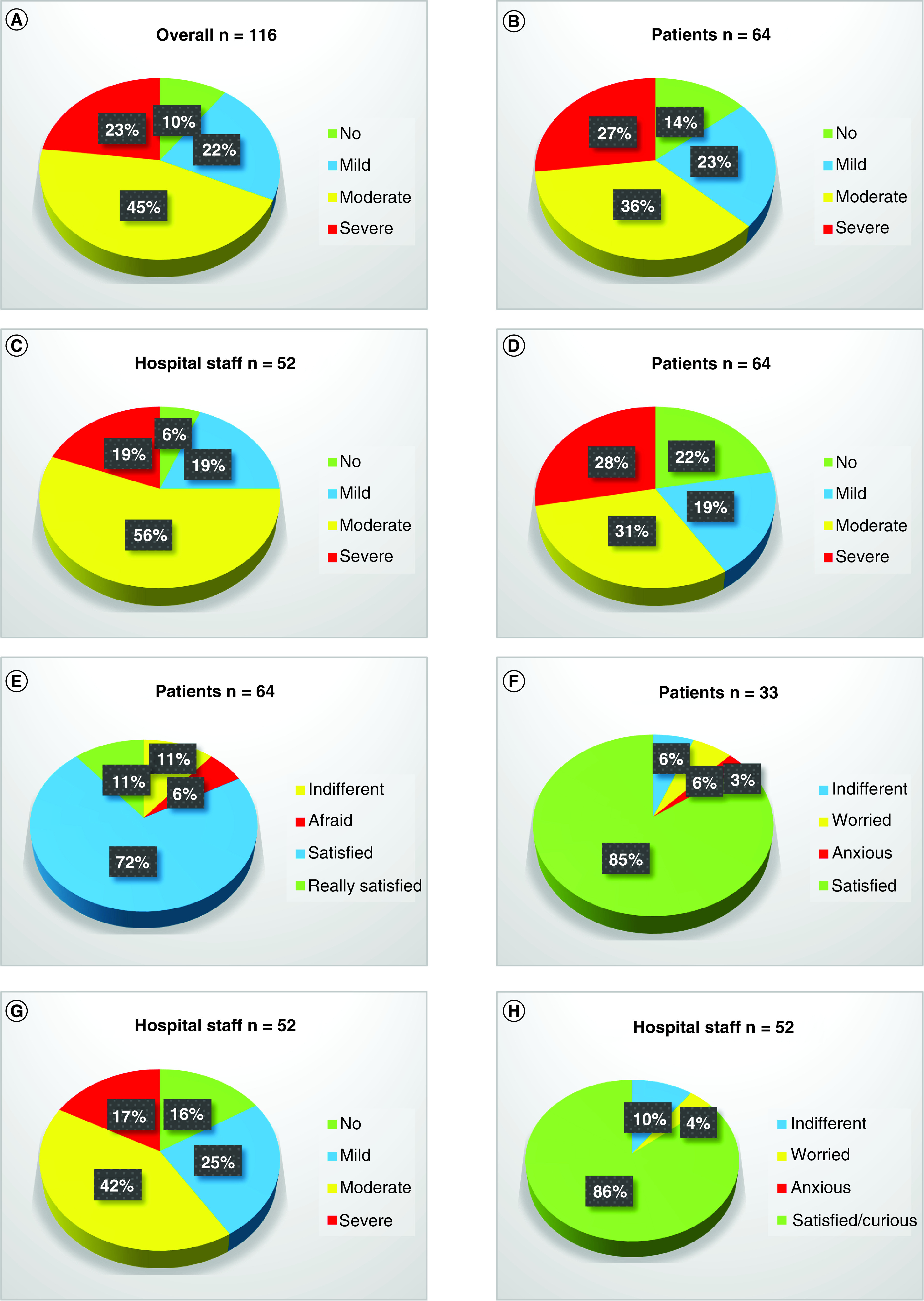
Pie charts representing the impact of the COVID-19 emergency on daily life and clinical practice. **(A)** How was your grade of concern because of the COVID-19 emergency? Overall. **(B)** How was your grade of concern because of the COVID-19 emergency? Patients. **(C)** How was your grade of concern because of the COVID-19 emergency? Hospital staff. **(D)** How was your grade of concern when physicians communicated to you the impossibility of performing surgery at your own center? Patients. **(E)** What was your reaction when physicians communicated to you the possibility of performing surgery at IEO? Patients. **(F)** What was your reaction when physicians communicated to you the possibility of performing surgery at IEO with a mixed hospital staff (H Niguarda-–IEO)? Patients from H Niguarda. **(G)** How was your grade of concern when your realized the necessity of carrying out your usual duties during the COVID-19 emergency? Hospital staff. **(H)** What was your reaction when you discovered a possible collaboration between H Niguarda hospital staff and IEO hospital staff? Hospital staff.

Of the 116 subjects who completed the survey, 68% reported a moderate/severe grade of concern due to the COVID-19 emergency (Figure 1A). Specifically, the percentage of subjects with a moderate/severe grade of concern due to the COVID-19 emergency was 63% in patients versus 75% in hospital staff members (Figure 1B & C). Of all patients treated during the study period (n = 64), 59% reported a moderate/severe grade of concern when referral clinicians communicated to them the impossibility of performing surgery at their own spoke centers (Figure 1D). However, 83% of them were satisfied/really satisfied when they discovered the possibility of performing surgery at the hub center (Figure 1E). Last, 85% of patients were satisfied when they discovered the possibility of shared management between the spoke and hub centers (Figure 1F). Of all hospital staff members (n = 52), 59% reported a moderate/severe grade of concern when they realized the necessity of performing their usual duties during the COVID-19 emergency (Figure 1G). However, 86% of them were satisfied/curious when they discovered a possible collaboration between spoke and hub centers (Figure 1H).

### Patient satisfaction after treatment at the hub center

Patient satisfaction after treatment was measured on a scale from 1 (minimum) to 5 (maximum). Overall, the grade of satisfaction after treatment at the hub center was 4 or 5 in 97% of patients. Specifically, the grade of satisfaction after treatment was 4 or 5 in 100 and 94%, respectively, in patients coming from spoke centers and managed exclusively by the hub center or the shared-management spoke–hub center. Last, 94% of patients were satisfied (grade 4 or 5) about the collaboration between spoke and hub staff members (Table 1).

**Table 1.** Patient satisfaction after treatment at the hub center.

Question	Scale (%)				
	1	2	3	4	5
Are you satisfied about treatment received at IEO center? Overall	0	1.5	1.5	18.5	78.5
Are you satisfied about treatment received at IEO center? Patients from other spoke centers	0	0	0	20	80
Are you satisfied about treatment received at IEO center? Patients from H Niguarda	0	3.0	3.0	18.5	75.5
Are you satisfied about the collaboration between hospital staff from H Niguarda and hospital staff from IEO? Patients from H Niguarda	0	0	6.0	12.5	81.5

A scale from 1 (minimum) to 5 (maximum) was used. Percentages of patients responding with each score are reported.

### Preventive strategies to reduce the risk of SARS-CoV-2 contagion

The adequacy of preventive strategies to reduce the risk of SARS-CoV-2 contagion was measured on a scale from 1 (minimum) to 5 (maximum). Overall, the grade of satisfaction about preventive strategies was 4 or 5 in 97% of patients. Specifically 97, 95.5, 97 and 97% of patients considered the preventive strategies adequate (level 4 or 5) at hospital entry, at ambulatories, at the urology department and at operating rooms, respectively. Last, 100% of patients considered adequate (grade 4 or 5) all preventive strategies adopted by hospital staff members (Table 2).

**Table 2.** Level of satisfaction about preventive strategies to reduce the risk of SARS-CoV-2 contagion.

Question	Scale (%)				
	1	2	3	4	5
How was your grade of satisfaction about preventive strategies to reduce the risk of contagion at IEO? Patients	0	0	3.0	15.5	81.5
How was your grade of satisfaction about preventive strategies to reduce the risk of contagion at hospital entry? Patients	0	0	3.0	15.5	81.5
How was your grade of satisfaction about preventive strategies to reduce the risk of contagion at ambulatories? Patients	0	0	4.5	28.5	67.0
How was your grade of satisfaction about preventive strategies to reduce the risk of contagion at urologic department? Patients	0	0	3.0	15.5	81.5
How was your grade of satisfaction about preventive strategies to reduce the risk of contagion at operating rooms? Patients	0	0	3.0	17.0	80.0
How was your grade of satisfaction about preventive strategies to reduce the risk of contagion adopted by hospital staff? Patients	0	0	0	17.0	83.0
How was your grade of satisfaction about preventive strategies to reduce the risk of contagion adopted by patients? Hospital staff	1.5	15.0	51.0	23.0	9.5
How was your grade of satisfaction about preventive strategies to reduce the risk of contagion adopted by hospital staff? Hospital staff	13.0	29.0	39.0	13.0	6.0
How was your grade of satisfaction about preventive strategies to reduce the risk of contagion at ambulatories/urologic department? Hospital staff	11.0	9.5	37.5	36.0	6.0
How was your grade of satisfaction about preventive strategies to reduce the risk of contagion at operating rooms? Hospital staff	3.0	12.5	28.5	48.5	7.5

A scale from 1 (minimum) to 5 (maximum) was used. Percentages of patients responding with each score are reported.

Overall, 32.5 and 19% of hospital staff members considered preventive strategies adequate (grade 4 or 5) for patients and hospital staff. Last, 42 and 56% of hospital staff members considered preventive strategies adequate (level 4 or 5) at ambulatories/urology department and operative rooms.

### Collaboration between spoke & hub center staff

**Figure 2.**
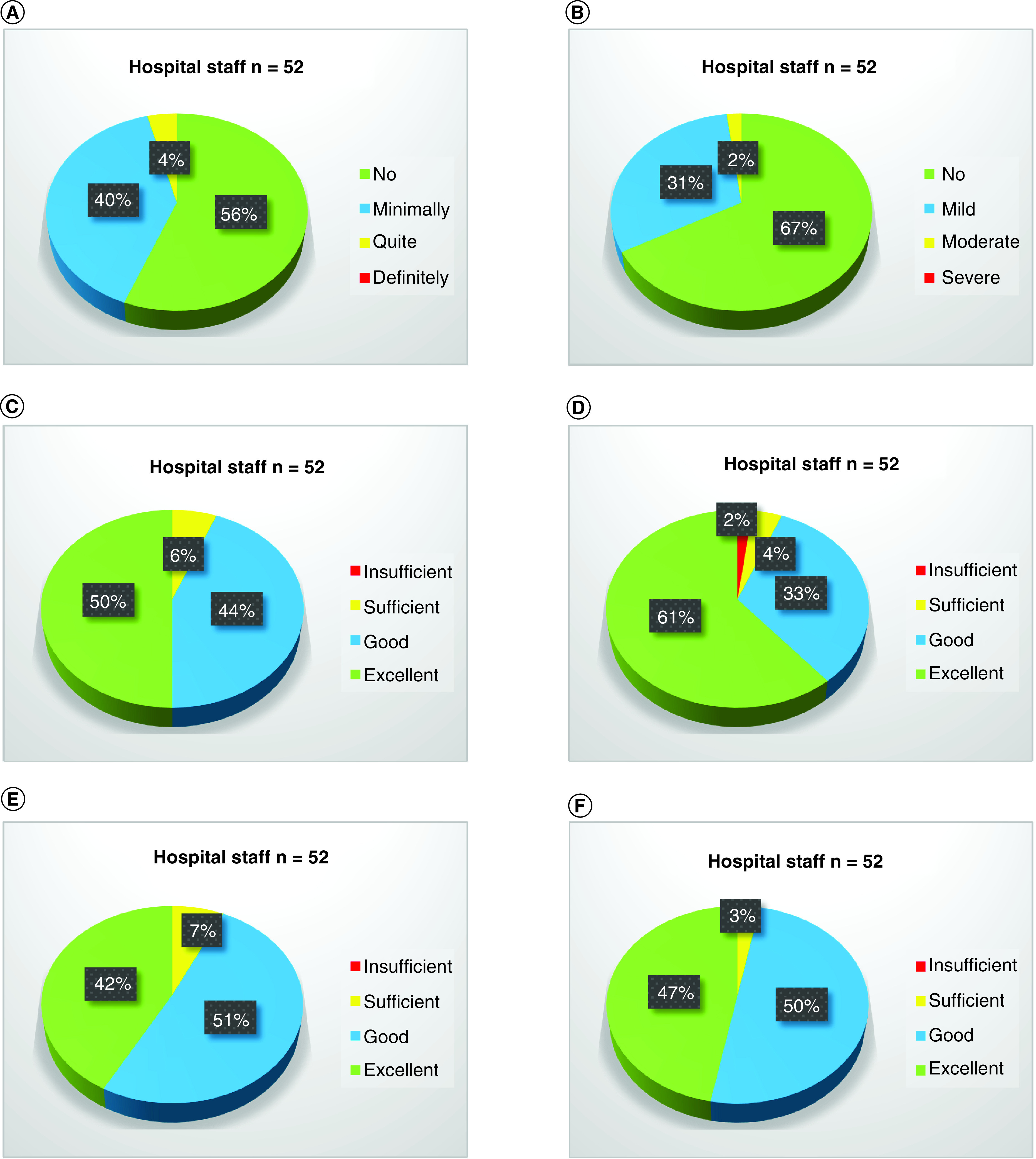
Pie charts representing the level of satisfaction about the collaboration between spoke and hub center staff. **(A)** How was your grade of discomfort during daily practice because of different work habits of colleagues from other hospitals? **(B)** How was your grade of anxiety when working with colleagues from other hospitals? **(C)** How was the level of co-operation between clinicians from H Niguarda and clinicians from IEO? **(D)** How was the level of co-operation between clinicians from H Niguarda and ward/scrub nurses from IEO? **(E)** How was the level of adaptation of clinicians from H Niguarda to different work habits at ambulatories/urology department? **(F)** How was the level of adaptation of clinicians from H Niguarda to different work habits in the operating rooms?.

Of all hospital staff members who completed the survey (n = 52), 96% reported no/minimal discomfort during daily practice because of different work habits of colleagues from the other hospital (Figure 2A). Moreover, 98% of them reported no/minimal anxiety when working with colleagues from other hospitals (Figure 2B). Overall, 94% of hospital staff members from the spoke center reported a good/excellent level of co-operation with either clinicians (Figure 2C) or ward/scrub nurses from the hub center (Figure 2D). Last, the level of adaptation of clinicians from the spoke center to different work habits at the ambulatories/urology department (Figure 2E) or operating room (Figure 2F) was reported as good/excellent in 93 and 97% respectively.

### Final considerations & model reproducibility

**Figure 3.**
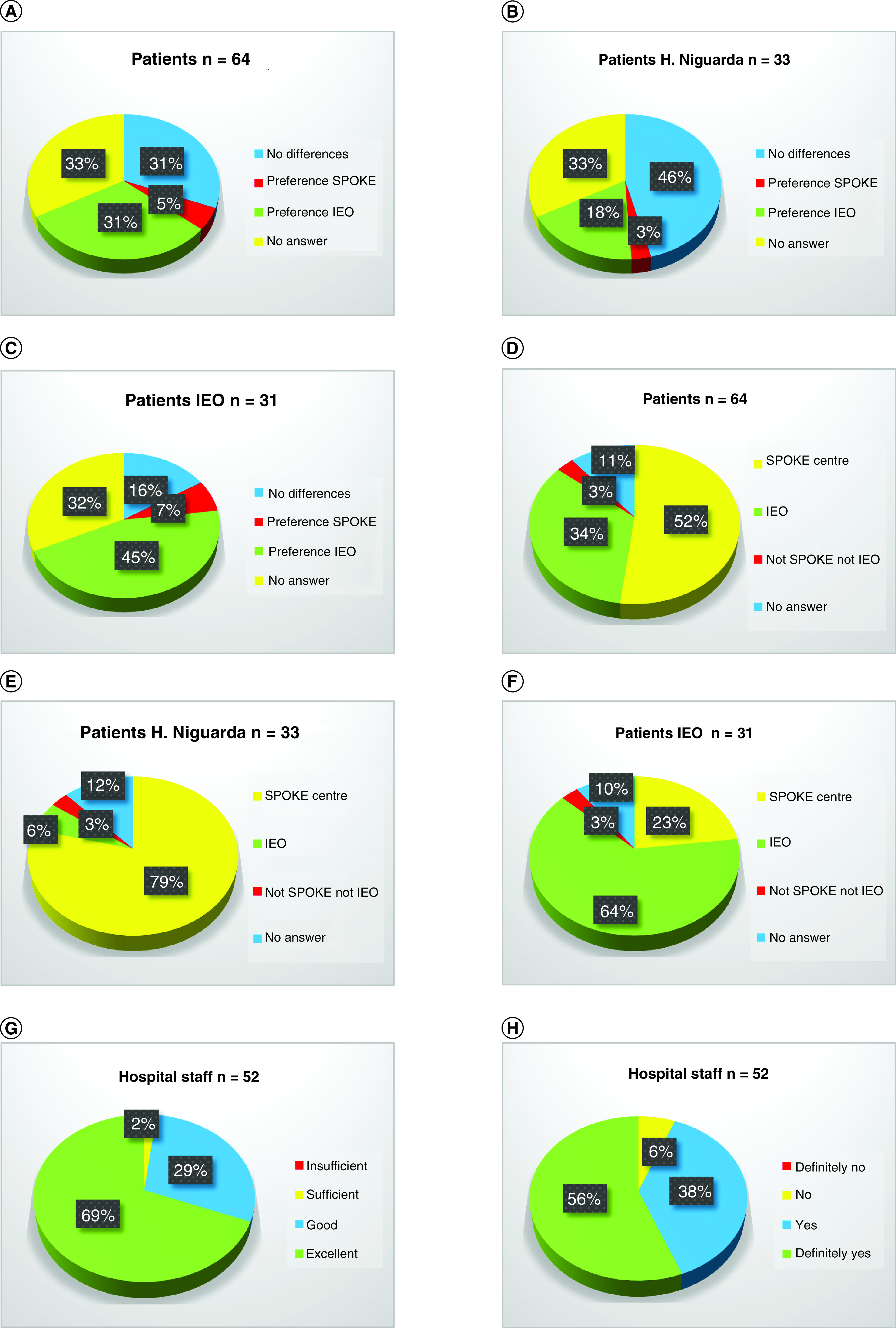
Pie charts representing final considerations. **(A)** Have you observed differences between your own center and IEO? Overall patients. **(B)** Have you observed differences between your own center and IEO? Patients from H Niguarda. **(C)** Have you observed differences between your own center and IEO? Patients from other spoke centers. **(D)** At which center are you going to perform follow-up visits after surgery? Overall patients. **(E)** At which center are you going to perform follow-up visits after surgery? Patients from H Niguarda. **(F)** At which center are you going to perform follow-up visits after surgery? Patients from other spoke centers. **(G)** How do you consider the experience ‘shared management of patients between spoke (H Niguarda) and hub center (IEO)’? Hospital staff. **(H)** Would you agree to repeat the experience ‘shared management of patients between spoke (H Niguarda) and hub center (IEO)’? Hospital staff. **(I)** Do you believe that this experience helped your professional training? Hospital staff. **(J)** Do you believe that this experience helped your clinical training? Clinicians. **(K)** Do you believe that this experience helped your surgical training? Clinicians. **(L)** Do you believe that this experience helped your scientific training? Clinicians. **(M)** Do you believe that this experience could have created the basis for collaboration between H Niguarda and IEO centers for clinical/scientific trials in the future? Clinicians.

Of all 64 patients who completed the survey, 31% reported no differences between the hub center and their original spoke centers (Figure 3A). Only 5% stated that they would have preferred to be treated at their own original spoke center. Conversely, 31% preferred to be treated at the hub center. The percentages of ‘no difference’ versus ‘preference: spoke’ versus ‘preference: hub’ were, respectively, 46 versus 3 versus 18% (Figure 3B) and 16 versus 7 versus 45% (Figure 3C) in patients managed by the shared-management spoke–hub center and in patients coming from other spoke centers and managed exclusively by the hub. Overall, 52 versus 34% of patients reported that they would perform follow-up visits at the spoke versus the hub center, respectively (Figure 3D). Specifically, follow-up visits would be performed in the spoke versus the hub center in 79 versus 6% (Figure 3E) and 23 versus 64% (Figure 3F), respectively, of patients managed by the shared-management spoke–hub center and those managed exclusively by the hub center.

Of all 52 hospital staff members who completed the survey, 98% considered good/excellent their experience of the shared management spoke–hub center (Figure 3G). Moreover, 94% of them would not have concerns in repeating this experience (Figure 3H). Overall, 66% of hospital staff members reported that this experience helped their professional training (Figure 3I). Specifically, 44, 40 and 24% of clinicians (n = 25) reported that this experience helped their clinical (Figure 3J), surgical (Figure 3K) and scientific (Figure 3L) training, respectively. Last, 88% of clinicians believed that this experience could have created the basis for collaboration between the spoke (H Niguarda) and hub (IEO) centers for clinical/scientific trials in the future (Figure 3M).

**Figure 4.**
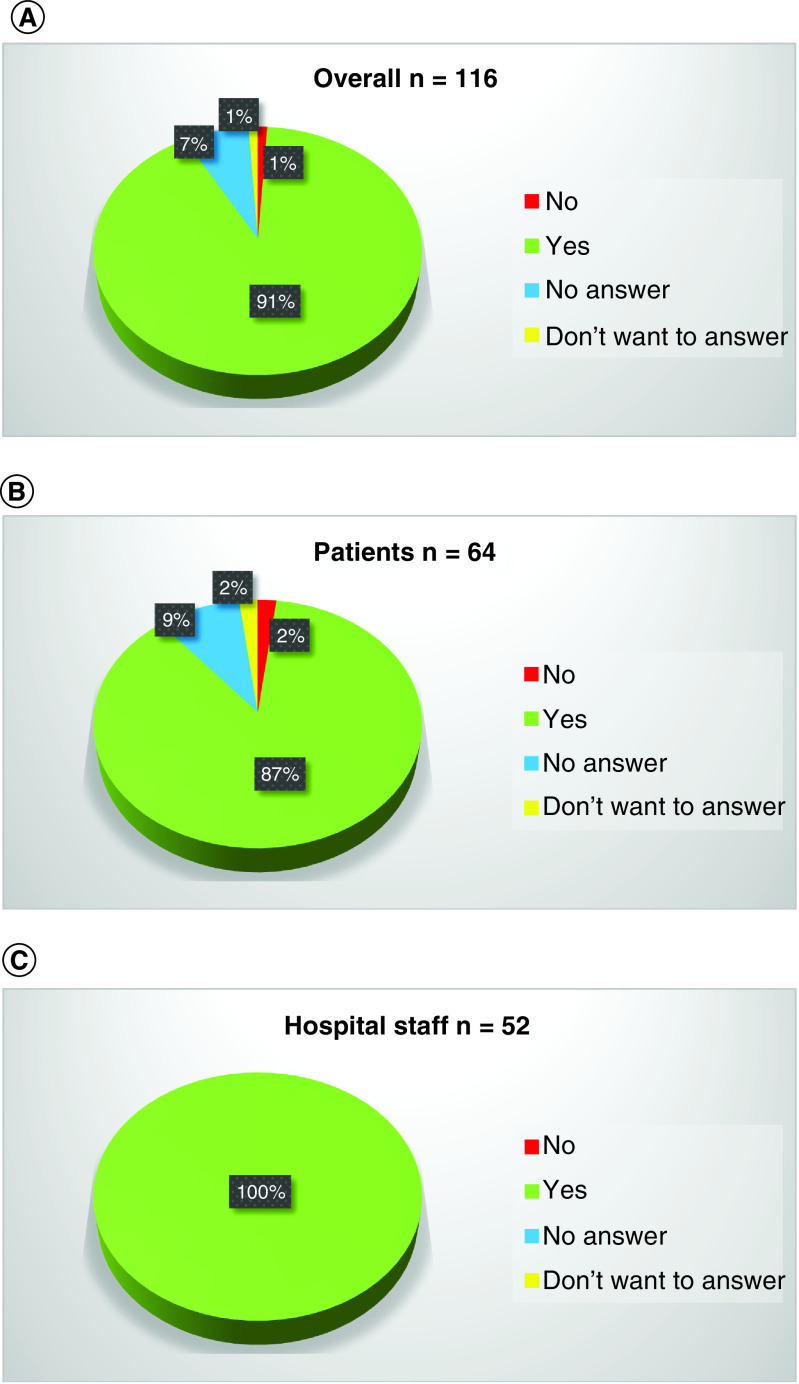
Pie charts representing model reproducibility. **(A)** Do you believe that the IEO center could represent a credible solution for management of non-deferrable uro-oncologic patients during the COVID-19 emergency? Overall. **(B)** Do you believe that the IEO center could represent a credible solution for management of non-deferrable uro-oncologic patients during the COVID-19 emergency? Patients. **(C)** Do you believe that the IEO center could represent a credible solution for management of non-deferrable uro-oncologic patients during the COVID-19 emergency? Hospital staff.

Last, 91% of subjects who completed the survey (n = 116) believed that the hub center could represent a credible solution for management of non-deferrable uro-oncologic patients during the COVID-19 emergency (Figure 4A). Specifically, 87 and 100% of patients (Figure 4B) and hospital staff members (Figure 4C), respectively, believed that this experience could be reproducible in the future.

## Discussion

Following our experience of creating an oncologic hub during the COVID-19 pandemic [6], where non-deferrable cancer patients could be safely managed and where patients coming from spoke centers could be treated with or without the collaboration of peers from those centers, we conducted a survey to test patients’ and healthcare professionals’ involvement and satisfaction with the model. Our results showed several important findings that could be useful for clinical practice and for healthcare system organization.

First, we observed a moderate/severe grade of concern due to the COVID-19 emergency in 63% of patients versus 75% of hospital staff members. A moderate/severe grade of concern was also recorded in 59% of patients due to the impossibility of performing surgery at their own original spoke centers. Moreover, 59% of hospital staff members reported a moderate/severe grade of concern due to the necessity of carrying out their job during the COVID-19 pandemic. Our results are consistent with several previous studies that addressed the psychosocial impact of the COVID-19 emergency across the different strata of society [8–10]. Specifically, Teoh *et al.* [11] reported that approximately 47% of urologists worldwide had expressed fear of going to work during the pandemic. Moreover, mass fear of COVID-19, named as ‘coronaphobia’, has had a universal psychosocial impact by causing mass hysteria, economic burden and financial losses [8]. Although we confirmed this enormous psychological impact caused by SARS-CoV-2 infection in our study population, we also observed a high grade of satisfaction, among both patients and hospital staff members, after discovering the possibility of performing surgery at the hub center. Moreover, high grades of satisfaction were also recorded in both categories (patients and hospital staff) after treatment.

To the best of our knowledge, we are the first to report psychosocial crisis prevention due to regional intervention models during the COVID-19 pandemic. Nonetheless, despite being promising, our results should be considered exploratory at best due to the small sample size of the current analysis. In consequence, future in-depth research activities should focus on specific questionnaires to evaluate the grade of discomfort due to the COVID-19 emergency in all surgical candidates. Moreover, specific psychological interventions targeting high-risk populations with heavy psychological distress due to the COVID-19 pandemic should be tested in future analyses.

Second, we analyzed the grade of satisfaction about preventive strategies to reduce the risk of SARS-CoV-2 contagion, as previously reported [6]. Here we observed different results in separate analyses that considered patients versus hospital staff members. Specifically, the grade of satisfaction was high/very high in the vast majority of patients, even after stratification of different hospital places, namely at hospital entry, ambulatories, urology department and operating rooms. Moreover, the totality of patients felt that all preventive strategies adopted by hospital staff members were adequate. The opposite results were observed in hospital staff members, among whom only 32.5 and 19% considered preventive strategies adequate for patients and hospital staff, respectively. Several explanations could justify these findings, although they appear consistent with a previous report by Teoh *et al.* [11] in which only 33% of the responders felt they were given adequate personal protective equipment. First, there are different degrees of knowledge in patients versus hospital staff members about medical devices and personal protective equipment available to reduce the risk of SARS-CoV-2 contagion [12–15]. Second, given that the study period covered approximately 4 months (16 March–1 June 2020), it is possible that preventive strategies recommended at the beginning of the COVID-19 pandemic may have been considered outdated when hospital staff members completed the survey. Third, it is possible that hospital staff members could have considered inadequate all preventive strategies adopted by the hub center compared with those of other hospitals involved in the management of COVID-19 patients. However, it has to be remembered that an oncologic hub center should be considered a ‘COVID-19 free hospital’, as previously reported [6]. The staff members’ concerns, in fact, might be considered over-rated in light also of the data that our operative protocol led to a very low rate of contagion inside the hub center, as previously described [6]. Fourth, due to their scarce availability at the beginning of the pandemic, individual protective devices and nasopharyngeal tests were prioritized to workers at higher risk of SARS-CoV-2 contagion. All the above explanations should be considered hypothetical at best. In consequence, despite several authors’ previously reported considerations and recommendations for preventing SARS-CoV-2 contagion in both patients [16–19] and healthcare workers [20–22], a more focused analysis on preventive strategies to reduce the risk of SARS-CoV-2 contagion at oncologic hub hospitals is warranted. For example, the European Association of Urology Guidelines Office Rapid Reaction Group recently advised to follow local recommendations for personal protective equipment distribution and for testing both staff and patients for COVID-19 during the pandemic [4,5].

Third, we reported a minimal grade of discomfort and excellent levels of co-operation between clinicians from the spoke center (H Niguarda) and hospital staff members of the hub center. Moreover, 98% of hospital staff members considered good/excellent their experience of the shared-management spoke–hub center. Additionally, 44, 40 and 24% of clinicians reported that this experience helped their clinical, surgical and scientific training, respectively. Last, the vast majority of clinicians believed in a possible collaboration between the spoke and hub centers for clinical/scientific trials in the future. In summary, these results indicate that shared management of patients and hospital resources during the COVID-19 emergency is well accepted by clinicians. Moreover, sharing knowledge and experience could permit the implementation of training programs and generate scientific collaborations not only during the pandemic era, but also during daily clinical practice.

Finally, we tested model reproducibility. Overall, we observed that 91% of subjects who completed the survey believed that the hub center could represent a credible solution for the management of non-deferrable uro-oncologic patients during the COVID-19 emergency. These results remained consistent in separate analyses that considered patients versus hospital staff members. Specifically, the vast majority of patients (87%) and all hospital staff members (100%) believed that this experience could be reproducible in the future.

Taken together, our findings provide robust evidence that an oncologic hub center during the COVID-19 pandemic represents a credible solution for management of non-deferrable uro-oncologic patients. Specifically, surgical treatment at the hub center of patients coming from spoke centers is well accepted by both patients and hospital staff members. Moreover, collaboration between healthcare workers from spoke and hub centers generates only minimal anxiety, while being associated with potential clinical, surgical and scientific improvement. This said, a more specific focus on recommended strategies to reduce the risk of SARS-CoV-2 contagion at oncologic hub hospitals is warranted.

Despite its novelty, our study has limitations. First, the current data are influenced by inherent selection bias. Moreover, partial sampling (70.5 vs 72% of patients in category 1 vs category 2) may result in systematic biases and residual confounding relative to the entire population. Second, as previously stated, we lacked validated questionnaires to evaluate the psychological impact of the COVID-19 emergency on both patients and hospital staff members. Third, the current analysis is monocentric and limited by the small number of patients treated. However, this study represents a real-life scenario. Moreover, only one spoke center (H Niguarda) was involved in the shared-management spoke–hub center program. In consequence, data from other oncologic hub centers are urgently needed. Fourth, follow-up data on long-term oncologic outcomes are lacking. Last, information about the number of SARS-CoV-2-infected patients during their hospital stay and immediately after discharge was unavailable. In consequence, a specific focus on the risk of COVID-19 infection in hub hospitals is warranted.

## Conclusion

Oncologic hub centers represent a credible and well-accepted solution and should therefore be recommended for the management of all non-deferrable uro-oncologic patients during the COVID-19 pandemic. However, implementation of preventive strategies to reduce the risk of SARS-CoV-2 contagion among hospital staff members is warranted. Last, follow-up data from other regional intervention models during the COVID-19 emergency are urgently needed

## Future perspective

Oncologic hub centers will provide a credible solution during future pandemic.

**Summary points**

- Treatment at an oncologic hub during COVID-19 pandemic was well accepted by patients.
- Preventive strategies to reduce the risk of SARS-CoV-2 contagion were considered satisfactory by patients.
- Preventive strategies to reduce the risk of SARS-CoV-2 contagion were considered insufficient by hospital staff.
- Collaboration between spoke center and hub center staff is excellent.
