# Supplementary material for: Uro-oncologic patient management during the COVID-19 pandemic: survey findings from an Italian oncologic hub
Source: Future Oncol. 2021 Jul 19:10.2217/fon-2021-0145. doi: 10.2217/fon-2021-0145 (PMC8288285; doi:10.2217/fon-2021-0145)
Supplement: Supplementary file 1 [file Supplementary_Figure_1.pdf]

IEO
Istituto Europeo di Oncologia

Cognome:

Nome:

Data di nascita:

Intervento eseguito:

1) **In generale, quale era il suo livello di preoccupazione a causa dell'emergenza COVID-19?** (cerchiare/barrare la risposta)
a) Nessuna
b) Poco
c) Abbastanza
d) Molto

2) **Quale era il suo livello di preoccupazione quando le hanno comunicato che non poteva eseguire l'intervento chirurgico programmato presso il suo centro di cura?** (cerchiare/barrare la risposta)
a) Nessuna
b) Poco
c) Abbastanza
d) Molto

3) **Quando le hanno comunicato la possibilità di eseguire l'intervento chirurgico programmato presso il centro IEO, come ha reagito?** (cerchiare/barrare la risposta)
a) Indifferente. L'importante è eseguire l'intervento chirurgico.
b) Spaventato/a. Avrei preferito aspettare la fine dell'emergenza COVID-19 ed eseguire l'intervento presso il centro in cui ero in cura (Osp. Niguarda).
c) Soddisfatto/a. Ho capito la necessità del momento e sono contento/a del fatto di non essere stato trascurato/a.
d) Ampiamento soddisfatto/a. Preferivo essere trattato/a presso lo IEO piuttosto che nel mio centro dove ero in cura (Osp. Niguarda).

4) **Quando le hanno comunicato la possibilità di eseguire un intervento chirurgico presso il centro IEO con gestione a carico di equipe mista NIGUARDA/IEO, come ha reagito** (cerchiare/barrare la risposta)**?**
a) Indifferente. L'importante è eseguire l'intervento chirurgico.
b) Spaventato/a. Una equipe mista può generare confusione nella gestione del paziente. Avrei preferito essere gestito/a interamente da personale del mio ospedale (Niguarda).
c) Spaventato/a. Una equipe mista può generare confusione nella gestione del paziente. Avrei preferito essere gestito/a interamente da personale IEO.
d) Soddisfatto/a. Nonostante l'emergenza COVID-19, ero contento/a che i medici del mio ospedale (Niguarda) mi potessero seguire durante il ricovero.

5) **In una scala da 1 (minimo) a 5 (massimo), quanto si sente soddisfatto/a del trattamento chirurgico eseguito in IEO** (scrivere numero qui accanto)**:**

6) **In una scala da 1 (minimo) a 5 (massimo), quanto si sente soddisfatto/a della collaborazione tra i medici del suo ospedale (Niguarda) e lo staff sanitario IEO (medici, infermieri, operatori socio-sanitari)?** (scrivere numero qui accanto)**:**

7) **In una scala da 1 (minimo) a 5 (massimo), quanto reputa adeguate le misure anti-COVID adottate dallo IEO durante l'emergenza COVID-19** (scrivere numero qui accanto)**:**

8) **In una scala da 1 (minimo) a 5 (massimo), quanto reputa adeguate le misure di prevenzione all'ingresso presso l'ospedale anti-COVID (misurazione temperatura corporea, dotazione di guanti e mascherine, ingresso vietato ad accompagnatori etc.)?** (scrivere numero qui accanto)**:**

9) **In una scala da 1 (minimo) a 5 (massimo), quanto reputa adeguate le misure di prevenzione anti-COVID presso gli ambulatori (prericoveri, centri prelievi etc.)?** (scrivere numero qui accanto)**:**

10) **In una scala da 1 (minimo) a 5 (massimo), quanto reputa adeguate le misure di prevenzione anti-COVID adottate dal reparto di urologia (dotazione di guanti e mascherine, disinfezione, accesso limitato ai parenti etc.)?** (scrivere numero qui accanto)**:**

11) **In una scala da 1 (minimo) a 5 (massimo), quanto reputa adeguate le misure di prevenzione anti-COVID presso il blocco/sala operatoria in IEO (dotazione di guanti e mascherine, disinfezione etc)?** (scrivere numero qui accanto)**:**

12) **In una scala da 1 (minimo) a 5 (massimo), quanto reputa adeguate le misure di prevenzione anti-COVID adottate dal personale sanitario presso il reparto di urologia (medici/infermieri/operatori socio sanitari)?** (scrivere numero qui accanto)**:**

13) **Al termine della sua esperienza con il centro IEO** (cerchiare/barrare la risposta)

a) Non ho notato particolari differenze rispetto al mio centro di riferimento (osp. Niguarda)
b) Avrei comunque preferito essere trattato presso il mio centro di riferimento (osp. Niguarda) al termine dell'emergenza COVID-19
c) Ho preferito essere trattato in IEO rispetto al mio centro di riferimento (osp. Niguarda)
d) Non voglio esprimere un giudizio perché sarebbe influenzato dalla situazione di emergenza nazionale

14) **Al termine della sua esperienza con il centro IEO eseguirà i necessari controlli** (cerchiare/barrare la risposta)**:**

a) Tornerò al mio centro originario di cura (osp. Niguarda)
b) Continuerò il mio percorso presso il centro IEO visto che oramai sono stato/a operato/a presso questo ospedale
c) Mi affiderò a un centro che non sia né lo IEO né il mio centro originario di cura (osp. Niguarda)
d) Deciderò con calma dove proseguire eseguire i controlli al termine del periodo di emergenza COVID-19

15) Reputa che la soluzione "centro IEO come centro di riferimento oncologico durante la pandemia COVID-19" rappresenti una buona soluzione per il futuro, qualora si ripresentasse una simile situazione di emergenza? (cerchiare/barrare la risposta)

a) No

b) Si

c) Non sono in grado di rispondere a questa domanda

d) Non voglio rispondere a questa domanda
