# Supplementary material for: Uro-oncologic patient management during the COVID-19 pandemic: survey findings from an Italian oncologic hub
Source: Future Oncol. 2021 Jul 19:10.2217/fon-2021-0145. doi: 10.2217/fon-2021-0145 (PMC8288285; doi:10.2217/fon-2021-0145)
Supplement: Supplementary file 2 [file Supplementary_Figure_2.pdf]

Sesso:

Età:

Anni di lavoro totali:

Anni di lavoro presso IEO:

1) **In generale, quale era il tuo livello di preoccupazione a causa dell'emergenza COVID-19?** (cerchiare/barrare la risposta)
a) Nessuna
b) Poco
c) Abbastanza
d) Molto

2) **In generale, quale era il tuo livello di preoccupazione nel dover eseguire la tua professione durante l'emergenza COVID-19?** (cerchiare/barrare la risposta)
a) Nessuna
b) Poco
c) Abbastanza
d) Molto

3) **In generale, come hai reagito quando hai scoperto della collaborazione IEO/NIGUARDA durante il periodo COVID-19** (cerchiare/barrare la risposta)**?**
a) Indifferenza. Lavorare con medici di una equipe diversa non mi crea alcun problema.
b) Preoccupazione. Lavorare con medici di una equipe diversa e con diverse abitudini può generare confusione e difficoltà sul lavoro.
c) Ansia. Non ero sicuro/a di essere all'altezza del compito.
d) Soddisfazione/curiosità. Mi sembrava un' ottima occasione per aumentare il mio bagaglio di conoscenze e per testare la bontà del mio lavoro quotidiano.

4) **Come reputi il livello di adattamento dell'equipe Niguarda alle dinamiche di reparto/ambulatorio dell'urologia IEO (giro visite, sistemi informatici, terapie etc.)? (**cerchiare/barrare la risposta**):**
a) Insufficiente
b) Sufficiente
c) Buono
d) Ottimo

5) **Come reputi il livello di adattamento dell'equipe urologia Niguarda alle dinamiche del blocco operatorio IEO (sign-in, consegne a strumentisti ecc.)? (**cerchiare/barrare la risposta**):**

e) Insufficiente
f) Sufficiente
g) Buono
h) Ottimo

6) **Come reputi il livello di collaborazione tra l'equipe Niguarda e i medici dell'urologia IEO (**cerchiare/barrare la risposta**)?**
a) Insufficiente
b) Sufficiente
c) Buono
d) Ottimo

7) **Come reputi il livello di collaborazione tra l'equipe Niguarda e il personale infermieristico/operatori socio sanitari dell'urologia IEO (**cerchiare/barrare la risposta**)?**
a) Insufficiente
b) Sufficiente
c) Buono
d) Ottimo

8) **Quale livello di stress/preoccupazione avevi a confrontarti con medici non del tuo ospedale (Osp. Niguarda) (**cerchiare/barrare la risposta**)?**
a) Nessuno
b) Lieve
c) Abbastanza
d) Molto

9) **Reputi che le diverse abitudini dei colleghi Niguarda ti abbiano creato problemi nel tuo lavoro quotidiano** (cerchiare/barrare la risposta**)?**
a) Assolutamente no. L'attività si è volta come nella normale routine IEO.
b) In minima parte. Trascorso il periodo di adattamento, l'attività si è svolta regolarmente.
c) Abbastanza. Le abitudini di colleghi Niguarda erano molto diverse, ma con calma ho saputo affrontarle.
d) Assolutamente si. Le abitudini dei colleghi Niguarda erano totalmente differenti ed è stato per me molto complesso adattare il mio lavoro quotidiano alle loro richieste.

10) **Reputi che le diverse abitudini dei colleghi Niguarda abbiano aiutato la tua formazione/ aumentato il tuo bagaglio di conoscenze** (cerchiare/barrare la risposta)**?**
a) No
b) In minima parte
c) Abbastanza
d) In maniera importante

11) **In una scala da 1 (minimo) a 5 (massimo), quanto reputi adeguate le misure di prevenzione anti-COVID adottate dallo IEO nei confronti dei pazienti? (**scrivere numero qui accanto)**:**

12) **In una scala da 1 (minimo) a 5 (massimo), quanto reputi adeguate le misure di prevenzione anti-COVID adottate dallo IEO nei confronti degli operatori sanitari (medici/infermieri/operatori socio sanitari)? (**scrivere numero qui accanto)**:**

13) In una scala da 1 (minimo) a 5 (massimo), quanto reputi adeguate le misure di prevenzione anti-COVID adottate dal reparto/ambulatori di urologia? (scrivere numero qui accanto)**:**

14) In una scala da 1 (minimo) a 5 (massimo), quanto reputi adeguate le misure di prevenzione anti-COVID adottate dal blocco operatorio? (scrivere numero qui accanto)**:**

15) Come consideri questa esperienza di gestione del paziente condiviso NIGUARDA-IEO durante il periodo COVID (una volta terminata l'esperienza)? (cerchiare/barrare la risposta)

a) Pessima. La gestione del paziente è stata troppo confusionaria.
b) Sufficiente. La collaborazione si è ridotta al minimo indispensabile.
c) Buona. Soddisfacente nella maggior parte dei casi, ma a volte ci sono stati dei problemi.
d) Ottima. Il rapporto di lavoro è stato eccellente nonostante le diversità di metodologia di lavoro.

16) Ti sentiresti di ripetere una simile esperienza (cerchiare/barrare la risposta)**?**

a) Assolutamente no.
b) No. Devono cambiare molti aspetti organizzativi.
c) Si. Esistono comunque degli aspetti migliorabili.
d) Assolutamente si.
