# Supplementary material for: Uro-oncologic patient management during the COVID-19 pandemic: survey findings from an Italian oncologic hub
Source: Future Oncol. 2021 Jul 19:10.2217/fon-2021-0145. doi: 10.2217/fon-2021-0145 (PMC8288285; doi:10.2217/fon-2021-0145)
Supplement: Supplementary file 3 [file Supplementary_Figure_3.pdf]

IEO
Istituto Europeo di Oncologia

Sesso:

Età:

Anni di lavoro totali:

Anni di lavoro presso Ospedale Niguarda/IEO:

1) **In generale, quale era il tuo livello di preoccupazione a causa dell'emergenza COVID-19?** (cerchiare/barrare la risposta)
a) Nessuna
b) Poco
c) Abbastanza
d) Molto

2) **In generale, quale era il tuo livello di preoccupazione nel dover eseguire la tua professione durante l'emergenza COVID-19?** (cerchiare/barrare la risposta)
a) Nessuna
b) Poco
c) Abbastanza
d) Molto

3) **In generale, come hai reagito quando hai scoperto della collaborazione IEO/NIGUARDA durante il periodo COVID-19** (cerchiare/barrare la risposta)**?**
a) Indifferenza. Lavorare con medici/infermieri di una equipe diversa non mi crea alcun problema.
b) Preoccupazione. Lavorare con medici/infermieri di una equipe diversa e con diverse abitudini può generare confusione e difficoltà sul lavoro.
c) Ansia. Non ero sicuro/a di essere all'altezza del compito.
d) Soddisfazione/curiosità. Mi sembrava un' ottima occasione per aumentare il mio bagaglio di conoscenze e per testare la bontà del mio lavoro quotidiano.

4) **Come reputi il livello di adattamento dell'equipe Niguarda alle dinamiche di reparto/ambulatorio dell'urologia IEO (giro visite, sistemi informatici, terapie etc.)? (**cerchiare/barrare la risposta**):**
a) Insufficiente
b) Sufficiente
c) Buono
d) Ottimo

5) **Come reputi il livello di adattamento dell'equipe Niguarda alle dinamiche del blocco operatorio IEO (sign-in, consegne a strumentisti etc.)? (**cerchiare/barrare la risposta**):**
a) Insufficiente
b) Sufficiente
c) Buono
d) Ottimo

6) Come reputi tuo livello di collaborazione tra l'equipe Niguarda e i medici dell'urologia IEO (cerchiare/barrare la risposta)**?**

a) Insufficiente
b) Sufficiente
c) Buono
d) Ottimo

7) Come reputi il livello di collaborazione tra l'equipe Niguarda e il personale infermieristico/operatori socio sanitari dell'urologia IEO (cerchiare/barrare la risposta)**?**

a) Insufficiente
b) Sufficiente
c) Buono
d) Ottimo

8) Quale livello di stress/preoccupazione avevi a confrontarti con medici/infermieri/operatori socio-sanitari non del tuo ospedale (cerchiare/barrare la risposta)**?**

a) Nessuno
b) Lieve
c) Abbastanza
d) Molto

9) Reputi che le diverse abitudini dei colleghi IEO/NIGUARDA ti abbiano creato problemi nel tuo lavoro quotidiano (cerchiare/barrare la risposta)**?**

a) Assolutamente no. L'attività si è svolta come nella normale routine.
b) In minima parte. Trascorso il periodo di adattamento, la mia attività si è svolta regolarmente senza subire rallentamenti.
c) Abbastanza. Le abitudini di colleghi IEO erano molto diverse, per cui ho dovuto prestare molto del mio tempo cercando di entrare nei meccanismi.
d) Assolutamente si. Le abitudini dei colleghi IEO erano totalmente differenti ed è stato per me molto complesso adattare il mio lavoro quotidiano alle loro esigenze.

10) Reputi che le diverse abitudini dei colleghi IEO/NIGUARDA abbiano aiutato la tua formazione/ aumentato il tuo bagaglio di conoscenze generali (cerchiare/barrare la risposta)**?**

a) No
b) In minima parte
c) Abbastanza
d) In maniera importante

IEO
Istituto Europeo di Oncologia

11) Reputi che le diverse abitudini dei colleghi IEO/NIGUARDA abbiano aiutato la tua formazione/ aumentato il tuo bagaglio di conoscenze in merito alla gestione clinica/di reparto dei pazienti (cerchiare/barrare la risposta)**?**

a) No
b) In minima parte
c) Abbastanza
d) In maniera importante

12) Reputi che le diverse abitudini dei colleghi IEO/NIGUARDA abbiano aiutato la tua formazione/ aumentato il tuo bagaglio di conoscenze chirurgiche urologiche (cerchiare/barrare la risposta)**?**

a) No
b) In minima parte
c) Abbastanza
d) In maniera importante

13) Reputi che le diverse abitudini dei colleghi IEO/NIGUARDA abbiano aiutato la tua formazione/ aumentato il tuo bagaglio di conoscenze di ricerca scientifica (cerchiare/barrare la risposta)**?**

a) No
b) In minima parte
c) Abbastanza
d) In maniera importante

14) In una scala da 1 (minimo) a 5 (massimo), quanto reputi adeguate le misure di prevenzione anti-COVID adottate dallo IEO nei confronti dei pazienti? (scrivere numero qui accanto)**:**

15) In una scala da 1 (minimo) a 5 (massimo), quanto reputi adeguate le misure di prevenzione anti-COVID adottate dallo IEO nei confronti degli operatori sanitari (medici/infermieri/operatori socio sanitari)? (scrivere numero qui accanto)**:**

16) In una scala da 1 (minimo) a 5 (massimo), quanto reputi adeguate le misure di prevenzione anti-COVID adottate dal reparto/ambulatori di urologia? (scrivere numero qui accanto)**:**

17) In una scala da 1 (minimo) a 5 (massimo), quanto reputi adeguate le misure di prevenzione anti-COVID adottate dal blocco operatorio? (scrivere numero qui accanto)**:**

18) Come consideri questa esperienza di gestione del paziente condiviso NIGUARDA-IEO durante il periodo COVID (una volta terminata l'esperienza)? (cerchiare/barrare la risposta)

a) Pessima. La gestione del paziente è stata troppo confusionaria.
b) Sufficiente. La collaborazione si è ridotta al minimo indispensabile.
c) Buona. Soddisfacente nella maggior parte dei casi, ma a volte ci sono stati dei problemi.
d) Ottima. Il rapporto di lavoro è stato eccellente nonostante le diversità di metodologia di lavoro.

IEO
Istituto Europeo di Oncologia

19) Ti sentiresti di ripetere una simile esperienza (cerchiare/barrare la risposta)**?**

a) Assolutamente no.
b) No. Devono cambiare molti aspetti organizzativi.
c) Si. Esistono comunque degli aspetti migliorabili.
d) Assolutamente si.

20) Al termine di questa esperienza, credi sia nata/possa nascere una collaborazione IEO-Niguarda per futuri studi clinici/scientifici/chirurgici (cerchiare/barrare la risposta)**?**

a) No. Credo che ognuno tornerà alla sua routine quotidiana.
b) Forse. Dipenderà da come sarà il carico di lavoro al termine dell'emergenza COVID-19
c) Si. In futuro coinvolgerò i colleghi IEO, qualora ce ne fosse occasione.
d) Assolutamente si. Ci stiamo già organizzando per condurre degli studi insieme.

21) Reputi che la soluzione "centro IEO come centro di riferimento oncologico durante la pandemia COVID-19" rappresenti una buona soluzione per il futuro, qualora si ripresentasse una simile situazione di emergenza? (cerchiare/barrare la risposta)

a) No
b) Si
c) Non sono in grado di rispondere a questa domanda
d) Non voglio rispondere a questa domanda
